# Hand Knob Syndrome Secondary to Ipsilateral Concomitant Carotid Fibromuscular Dysplasia and Proximal Atherosclerotic Disease

**DOI:** 10.7759/cureus.40072

**Published:** 2023-06-07

**Authors:** Mahmoud A Alshanqiti, Naif Alharbi, Faisal A Althobaiti, Saeed S Alzahrani, Mohammed Alwadai, Muhannad Asiri, Fawaz Alshareef, Mohammed Alqahtani, Turki F Bugshan

**Affiliations:** 1 Neurology, King Fahad Medical City, Riyadh, SAU; 2 Neurology, King Fahad General Hospital, Jeddah, SAU; 3 Neurology, Armed Forces Hospital - Southern Region, Khamis Mushait, SAU

**Keywords:** stroke, wrist drop, ica stent, fmd, hand knob stroke

## Abstract

One of the uncommon stroke presentations is the isolated wrist drop syndrome, caused by a stroke affecting the hand knob area, with the embolic mechanism being the most commonly identified mechanism. Here, we present the case of a 62-year-old female patient who presented with acute-onset isolated wrist drop secondary to right internal carotid artery fibromuscular dysplasia with a string of beads appearance and coexisting proximal atherosclerotic severe stenosis. The patient underwent successful carotid artery stenting. Patients with hand knob stroke may present a diagnostic dilemma and can be misdiagnosed as having peripheral neuropathy due to the absence of pyramidal signs and other symptoms of cortical involvement, leading to delayed or inappropriate treatment.

## Introduction

Stroke is a leading cause of death and disability worldwide. It is a complex and heterogeneous disease with various risk factors and clinical presentations. Among the less common presentations of stroke is the isolated wrist drop syndrome. This syndrome is caused by a stroke involving an isolated distal hand area (hand knob syndrome). This syndrome can result from various mechanisms such as small-vessel disease, cardioembolic, or arterial dissection [1]. In recent years, the incidence of this syndrome has increased, and several cases have been reported in the literature [2-6]. One potential mechanism is fibromuscular dysplasia (FMD) of the internal carotid artery (ICA), a rare vascular disease characterized by abnormal growth of the arterial wall [7,8]. Here, we present the case of a stroke patient who presented with hand knob syndrome and was found to have concomitant atherosclerotic stenosis as well as FMD involving the right ICA. The patient underwent successful right ICA stent deployment.

## Case presentation

**Figure 1:**
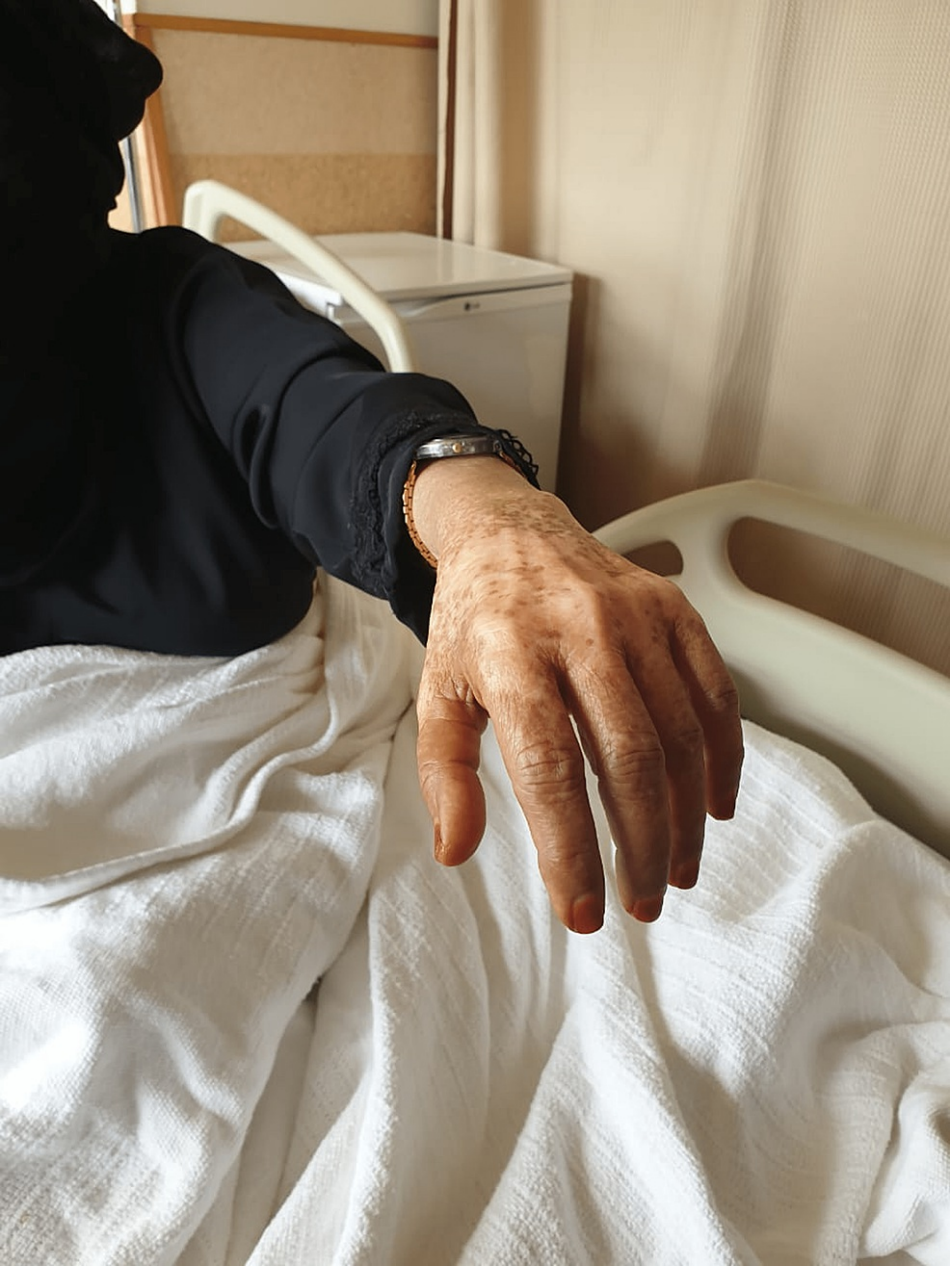
Isolated left wrist extension weakness (wrist drop).

A 62-year-old female patient, not known to have any medical illnesses or vascular risk factors, presented to our hospital with a history of sudden-onset isolated left-hand weakness of three days duration, with normal sensation. Her clinical examination revealed severe motor deficits in the left hand. The Medical Research Council (MRC) scale examination revealed grade 2 in the left wrist flexors, grade 1 in the left wrist extensors, grade 2 in the left finger flexors, grade 2 in the left finger extensors, and grade 3 in the left finger adduction and abduction (Figure 1). Left elbow flexion and extension along with left shoulder abduction and adduction were normal. There was mild hyperreflexia of the left biceps tendon reflex. Muscle power and coordination were normal in the left leg and right side of the body. Cranial nerves were normal as well.

**Figure 2:**
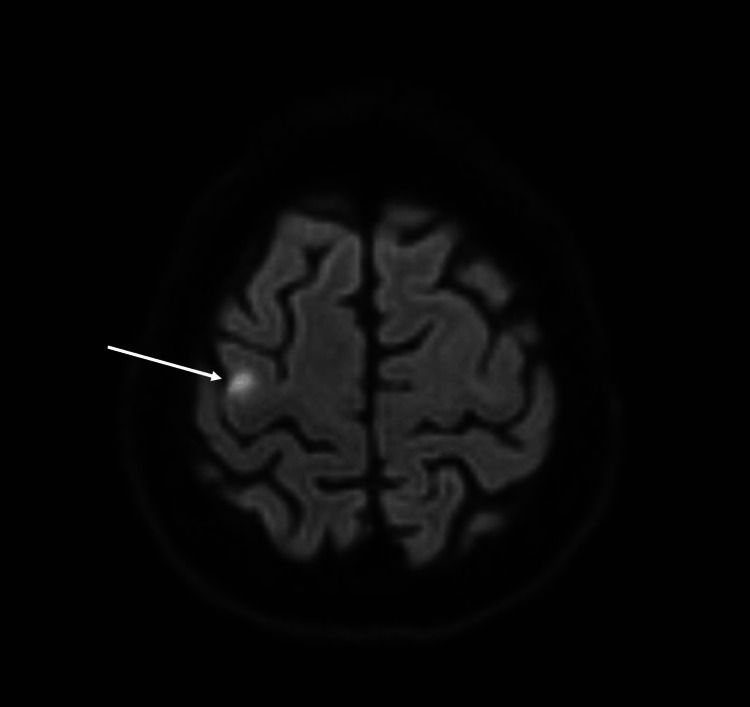
Magnetic resonance imaging diffusion-weighted imaging sequence of the brain showing an acute infarction involving the right precentral gyrus.

**Figure 3:**
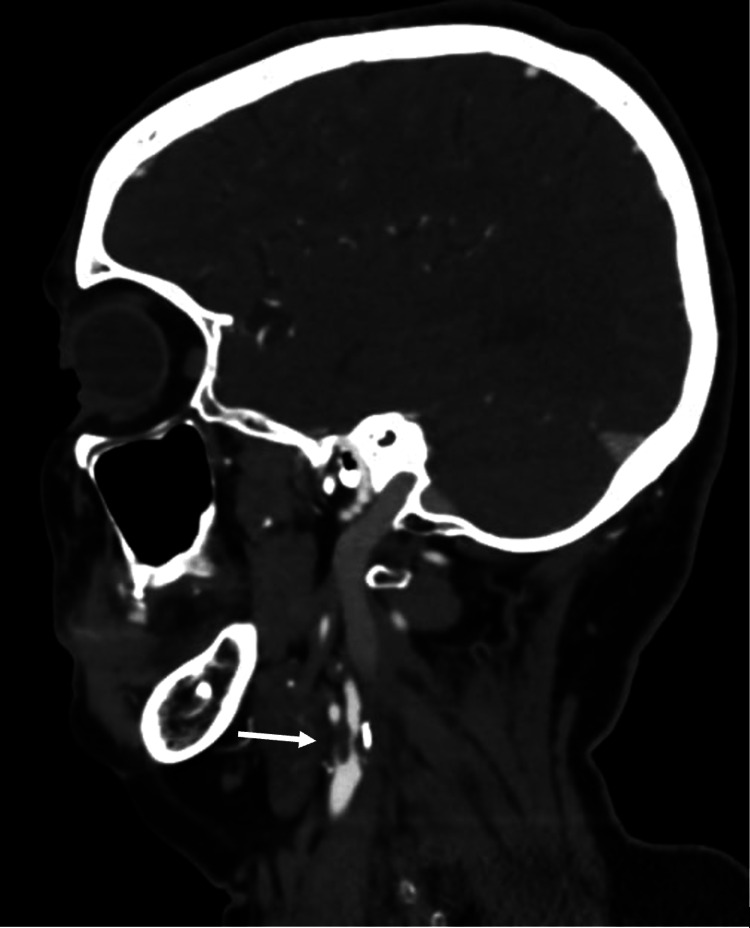
Computed tomography angiography showing severe luminal stenosis of around 90% involving the proximal segment of the right internal carotid artery associated with calcified plaque.

A complete stroke workup was subsequently performed. Magnetic resonance imaging (MRI) diffusion-weighted imaging (DWI) sequence of the brain confirmed the presence of acute infarction involving the right precentral gyrus (Figure 2). Carotid duplex showed severe right proximal ICA stenosis with peak systolic velocities of 450. Computed tomography (CT) angiography showed severe luminal narrowing of around 90% involving the proximal segment of the right ICA associated with calcified plaque (Figure 3); however, there was no evidence of intracranial vessel stenosis. Other routine blood work showed an abnormal lipid profile with total cholesterol of 220 mg/dL, high-density lipoprotein of 40 mg/dL, low-density lipoprotein of 150 mg/dL, and HbA1c of 6.2%. Cardiac workup was normal including frequent electrocardiograms, transthoracic echocardiogram, as well as Holter which showed no significant rhythm abnormalities.

**Figure 4:**
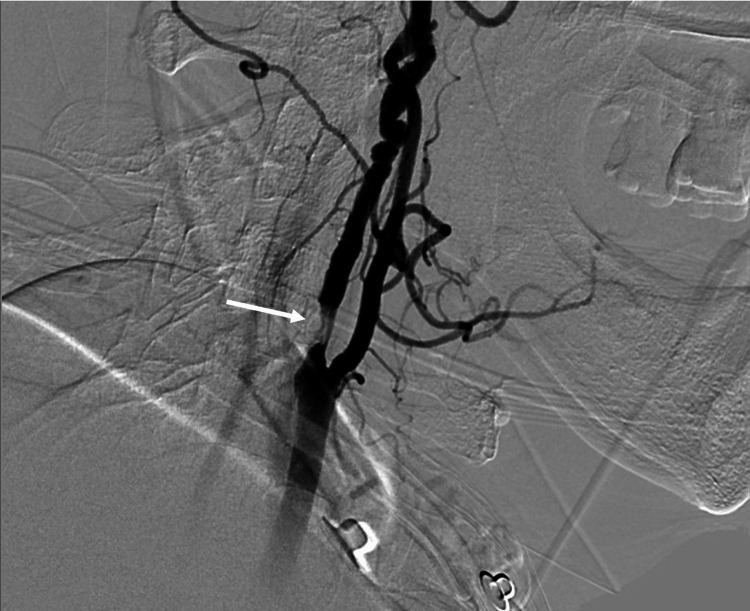
Conventional cerebral angiogram showing evidence of short segment, about 7.3 cm, and 90% luminal stenosis of the right proximal internal carotid artery.

**Figure 5:**
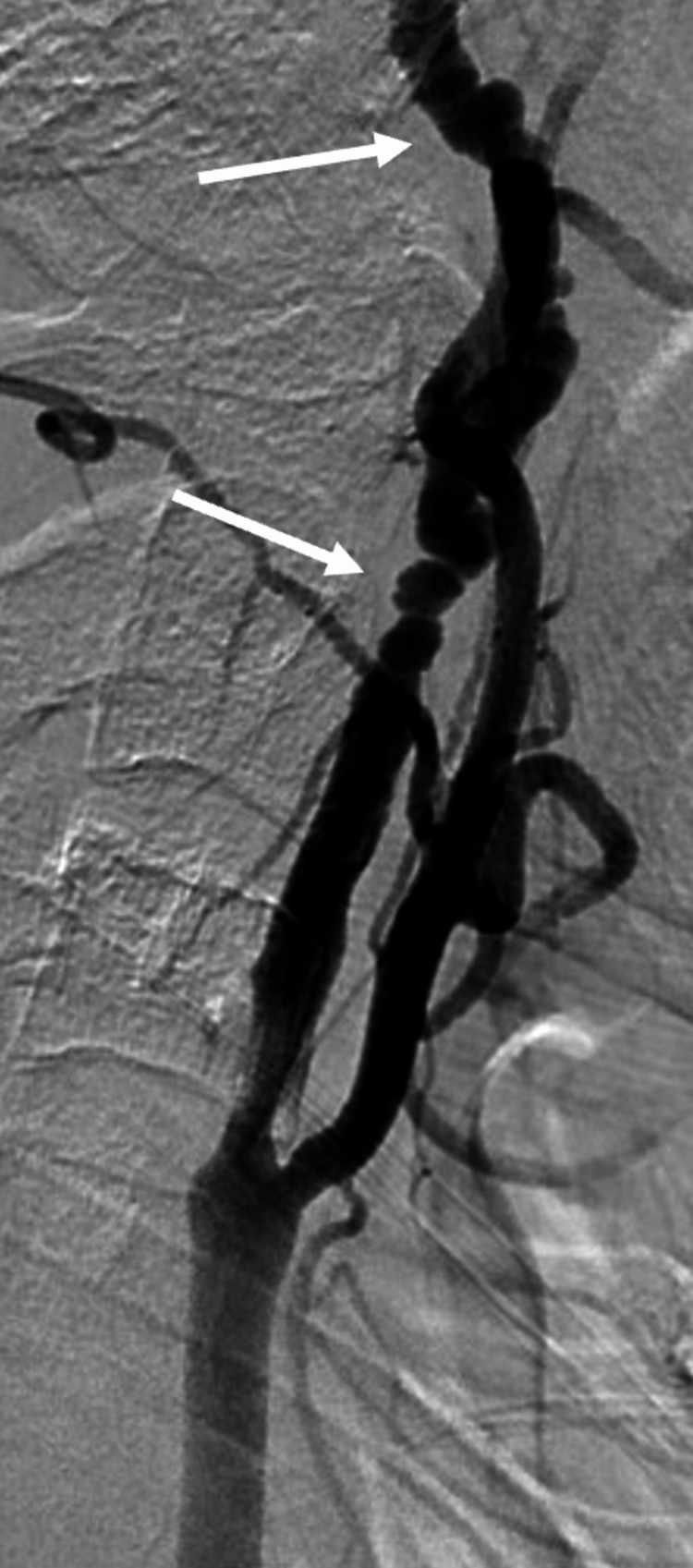
Conventional cerebral angiogram showing multiple characteristic arterial strings of beads in the petrous and lacerum parts of the right internal carotid artery.

A conventional cerebral angiogram revealed evidence of a short segment, about 7.3 cm, 90% luminal stenosis of the right proximal ICA, and multiple characteristic arterial strings of beads in the petrous and lacerum parts of the artery suggesting an underlying FMD (Figures 4, 5).

**Figure 6:**
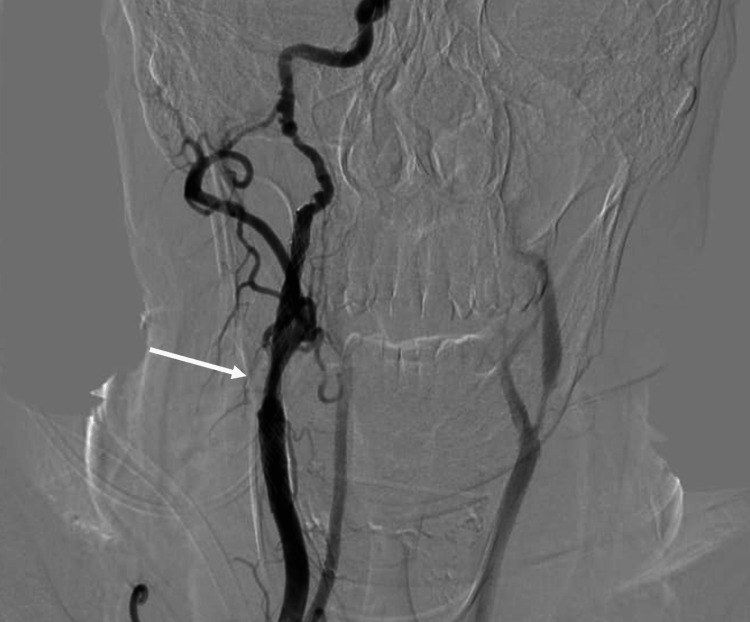
The Casper stent system successfully inserted through the stenotic segment.

**Figure 7:**
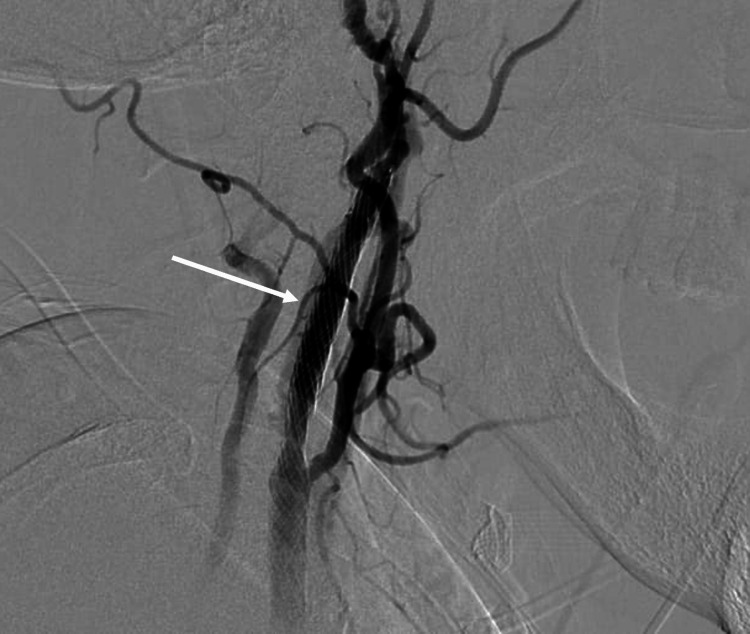
The Casper stent system successfully inserted through the stenotic segment achieving 85% balloon dilatation.

The Casper stent system was successfully inserted through the stenotic segment after initial balloon dilatation until 85% dilatation was achieved with no complications (Figures 6, 7). The patient remained neurologically intact throughout the procedure and was started on a dual antiplatelet regimen. She had an uneventful hospital course. Her follow-up duplex done after three months continued to show patent flow in the right carotid system with no evidence of stenosis. A renal duplex examination done later showed no evidence of FMD involving renal arteries.

## Discussion

This case illustrates an uncommon manifestation of stroke characterized by isolated wrist drop, which was found to be associated with both atherosclerotic stenosis and FMD of the right ICA.

Isolated wrist drop or hand knob stroke is an uncommon type of stroke with an incidence of less than 1% [1]. The hand knob area is a cortical site in the precentral gyrus that governs hand motor function. It was initially identified using functional MRI in 1997 and serves as a reliable anatomical reference for locating the precentral gyrus. Isolated hand palsy is typically considered a presentation of peripheral neuropathy [2], in addition to the absence of pyramidal signs and other symptoms of cortical involvement in patients with hand knob stroke, which may lead to misdiagnosis as peripheral neuropathy, causing delayed or inappropriate treatment [3].

In our case, the stroke mechanism of the patient’s cortical infarct was due to an ipsilateral artery-to-artery embolism secondary to carotid artery disease. A case series of hand knob strokes reported by Alstadhauga et al. concluded that more than two-thirds of hand knob strokes were due to embolic etiology, which is similar to another series that labeled the stroke mechanism as an embolic stroke of undetermined source [4,5]. Another study reported that more than 35% of hand knob stroke patients have more than 50% ipsilateral carotid artery stenosis [6]. To our knowledge, there are no reported cases of hand knob strokes secondary to FMD yet.

FMD affects the muscular walls of small-to-medium-sized arteries, causing non-inflammatory and non-atherosclerotic segmental lesions that lead to stenosis. It can either be symptomatic or clinically silent, and the lesions can be hemodynamically significant or not [7]. FMD can affect any arterial bed but is most commonly observed in the renal and extracranial carotid and vertebral arteries (in approximately 65% of cases) [8].

FMD can be classified based on the American Heart Association Classification of Fibromuscular Dysplasia into the multifocal type, which is the most common type displaying the beads-on-a-string appearance due to multiple stenoses. Unifocal FMD has long concentric stenosis [9]. In our case, the patient had localized extracranial carotid artery FMD of a multifocal subtype giving the string of beads appearance, but, interestingly, had coexisting more proximal just above the carotid bifurcation severe atherosclerotic stenosis, which is not commonly seen, as it is reported in fewer than 20% of patients with FMD based on some reports [10,11].

The management of FMD is primarily determined by the clinical presentation and the anatomy of the affected artery. Conservative treatment is recommended for asymptomatic FMD of the carotid artery, while interventional management is typically required for patients with symptomatic FMD of the extracranial carotid artery, including surgical dilatation with or without endarterectomy for the carotid arteries. Percutaneous transluminal balloon angioplasty with or without the application of stents or cerebral protection devices is the most commonly used method [7,12].

When severe atherosclerosis occurs concurrently with FMD at the carotid bifurcation, it can present a challenging treatment dilemma. Although mechanical treatment of the offending lesion may be necessary to prevent cerebral ischemia, determining which of the two concurrent lesions is causing the symptoms may be difficult. There is a lack of data on the short- and long-term outcomes of endovascular treatment for carotid FMD. The available literature consists mainly of small case series and retrospective studies. However, modern carotid angioplasty techniques, combined with cerebral protection devices, have shown promising results with effective treatment and minimal neurological morbidity [7,13,14]. In our case, based on the patient’s preference and our interventionist’s experience, the patient underwent successful balloon angioplasty and stent deployment. She remained neurologically intact throughout the procedure and had an uneventful hospital course. Her follow-up duplex showed right carotid system patent flow with no evidence of stenosis.

## Conclusions

This case presents an unusual manifestation of stroke, where the patient exhibited an isolated wrist drop. The condition was found to be linked with both atherosclerotic stenosis and FMD of the right ICA. Hand knob stroke patients may not exhibit pyramidal signs or other symptoms of cortical involvement, which can result in misdiagnosis as peripheral neuropathy, potentially leading to inadequate or delayed treatment.

## References

[1] Zhang Z, Sun X, Liu X, Wang L, Zhu R (2022). Clinical features, etiology, and prognosis of hand knob stroke: a case series. BMC Neurol.

[2] Tahir H, Daruwalla V, Meisel J, Kodsi SE (2016). Pseudoradial nerve palsy caused by acute ischemic stroke. J Investig Med High Impact Case Rep.

[3] Celebisoy M, Ozdemirkiran T, Tokucoglu F, Kaplangi DN, Arici S (2007). Isolated hand palsy due to cortical infarction: localization of the motor hand area. Neurologist.

[4] Alstadhaug KB, Sjulstad A (2013). Isolated hand paresis: a case series. Cerebrovasc Dis Extra.

[5] Finkelsteyn AM, Saucedo MA, Miquelini LA (2019). Ischemic stroke of the "hand knob area": a case series and literature review. J Clin Neurosci.

[6] Orosz P, Szőcs I, Rudas G, Folyovich A, Bereczki D, Vastagh I (2018). Cortical hand knob stroke: report of 25 cases. J Stroke Cerebrovasc Dis.

[7] Gornik HL, Persu A, Adlam D (2019). First international consensus on the diagnosis and management of fibromuscular dysplasia. Vasc Med.

[8] Olin JW, Gornik HL, Bacharach JM (2014). Fibromuscular dysplasia: state of the science and critical unanswered questions: a scientific statement from the American Heart Association. Circulation.

[9] Ismail M, Al-Ageely TA, Alzerkani MA (2022). Extracranial carotid localized fibromuscular dysplasia: a case report and literature review. Surg Neurol Int.

[10] Stahlfeld KR, Means JR, Didomenico P (2007). Carotid artery fibromuscular dysplasia. Am J Surg.

[11] Al-Nouri O, Pan J, Mannava K (2019). Endovascular treatment of concomitant carotid fibromuscular dysplasia and atherosclerotic disease after failed open surgical treatment. Vasc Endovascular Surg.

[12] Touzé E, Oppenheim C, Trystram D (2010). Fibromuscular dysplasia of cervical and intracranial arteries. Int J Stroke.

[13] Olin JW, Sealove BA (2011). Diagnosis, management, and future developments of fibromuscular dysplasia. J Vasc Surg.

[14] Xu JP, Cao YJ, Xiao GD, Zhang CY, Shi JJ (2015). Angioplasty and stenting for a young stroke patient diagnosed as cerebrovascular fibromuscular dysplasia. Neuroimmunol Neuroinflammation.

